# Preparation of Biomass-Based Ester End-Capped Hyperbranched Poly(ether)s via Facile One-Pot Reaction and Their Performance as Non-Toxic Plasticizers

**DOI:** 10.3390/polym12040913

**Published:** 2020-04-15

**Authors:** Qiaoguang Li, Xugang Shu, Puyou Jia, Yonghong Zhou

**Affiliations:** 1 School of Chemistry and Chemical Engineering, Zhongkai University of Agriculture and Engineering, Guangzhou 510225, China; liqiaoguang8799@163.com; 2 Institute of Chemical Industry of Forest Products, Chinese Academy of Forestry (CAF), 16 Suojin North Road, Nanjing 210042, China; zyh@icifp.cn

**Keywords:** hyperbranched poly(ether)s, plasticizer, acute oral toxicity, polyvinyl chloride, one-pot reaction

## Abstract

The aim of this study was to develop a facile one-pot reaction for the synthesis of biomass-based hyperbranched poly(ether)s end-capped as acetate esters (BHE) for use as a sustainable, safe and feasible plasticizer for flexible poly(vinyl chloride) (PVC) materials. BHE is completely miscible with PVC but shows weaker plasticizing effect than dioctyl phthalate (DOP) (EΔ*Tg* value of BHE reaches 64.8%). PVC plasticized with BHE displays greater thermal stability than that of PVC or PVC plasticized with DOP materials. BHE improves the thermal stability and flexibility of PVC materials. As a plasticizer, BHE displays lower solvent extractability and greater volatilization resistance than DOP. Acute oral toxicity indicates that BHE has toxic doses of 5 g/kg, suggesting that BHE is non-toxic.

## 1. Introduction

Poly(vinyl chloride) (PVC) is a thermoplastic resin derived from radical polymerization of vinyl chloride, which is widely used in construction materials, such as pipes, fittings, panels, flexible roofing masks, wire and cable layers, because of its low cost and excellent performance [1]. Although PVC has many excellent properties, it cannot be processed without plasticization. The addition of plasticizer can improve the machinability and flexibility of PVC materials by decreasing the interaction force and softening temperature, reducing the melt viscosity and increasing the fluidity of PVC molecules [2]. The level of plasticizer in flexible PVC materials usually ranges between 30–60 parts per hundred PVC. These flexible PVC materials are used to produce packaging materials, artificial leather and toys. Recently, the worldwide production of plasticizer exceeded 6 million tons per year, and phthalates now account for 80% of the total output of all plasticizers [3]. The global plasticizer manufacturing market was worth $93.76 billion in 2019. It is expected to grow at a compound annual growth rate (CAGR) of 4.4% and reach $111.38 billion by 2023. Phthalates easily migrate from PVC products by volatilization and may be extracted by a variety of solvents, which causes environmental pollution and is a threat to human health.

Studies on the toxicity of phthalates have been carried out for more than half a century. The lethal dose 50 (LD_50_) of dioctyl phthalate (DOP) in rats and mice has been reported to be 25 g/kg and 30 g/kg, respectively [4]. Recent studies indicate that the daily intake of DOP by the human body cannot exceed 69 mg/kg, which is lower than the amount of DOP that migrates from plasticized polymer products. DOP can migrate from products via volatilization and enter the human body through human respiration [5]. It is estimated that 500,000 pounds of phthalates was released to the environment from US manufacturing facilities in 1997 [6]. 

In order to overcome the toxicity and poor migration resistance of phthalates, a series of alternative plasticizers were prepared. The strategies to prevent plasticizer migration from PVC products mainly include internal plasticized method such as covalently attaching plasticizer onto the PVC matrix [7]; making surface modifications such as gamma irradiation, ultraviolet irradiation and plasma treatment [8]; and adding nanoparticles or ionic liquid [9,10]. These alternative plasticizers include CO_2_-based polycarbonate [11], cardanol-based epoxidized plasticizers [1], epoxidized vegetable oil [12], glycerol/adipic acid hyperbranched poly(ester)s [13], and so on. Glycerol/adipic acid hyperbranched poly(ester)s are thermally stable, are fully compatible with a PVC matrix, provide effective plasticization at acceptable levels, display low migratory potential and do not accelerate degradative dehydrochlorination [13]. However, there are very few reports on the acute oral toxicity of the plasticizers for PVC. 

Developing bio-based products is highly important because of their low cost, reproducibility and biodegradability [14,15,16]. In this study, we synthesized biomass-based hyperbranched poly(ether)s end-capped as acetate esters (BHE) via a facile one-pot reaction using triethyl citrate as raw material. The obtained BHE, with a larger relative molecular mass and a higher branching degree, is a potential plasticizer that could overcome the shortcoming of the poor migration resistance of lower relative molecular mass plasticizers, such as triethyl citrate and phthalate plasticizers. The plasticization and migration resistance of PVC materials plasticized with BHE were investigated and compared with those of DOP. The plasticizing mechanism was explored with regards to the shift of infrared absorption peaks of ester groups and the generation of hydrogen bonds via fourier transform infrared (FT-IR). In addition, an acute oral toxicity test was carried out to detect the toxicity of BHE. This study, which combines a one-pot reaction and acute oral toxicity and performance tests of BHE, provides promising candidates for the design and production of biomass-based, environmentally friendly and non-toxic plasticizers to replace phthalate plasticizers. 

## 2. Materials and Methods 

### 2.1. Materials

Dioctyl phthalate (DOP), triethyl citrate, glycidol, methanol, acetic anhydride, chloroform, sodium hydroxide, magnesium sulfate and tetrahydrofuran (THF) were purchased from Shanghai Aladdin Biochemical Technology Co., Ltd., Shanghai, China. PVC paste resin (degree of polymerization (DP) = 1300 ± 100, K value = 69.7–73.0) was provided by All Hanwha, Korea. All chemicals were used as received without further purification.

### 2.2. Synthesis of Biomass-Based Hyperbranched Polyol (BHP) and Biomass-Based Hyperbranched Poly(ether)s End-Capped as Acetate Esters (BHE)

**Figure 1:**
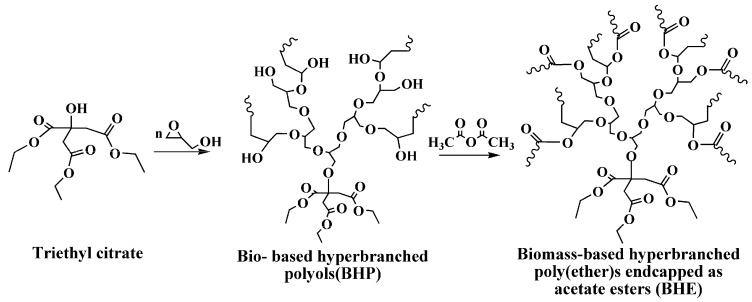
Synthesis of biomass-based hyperbranched poly(ether)s end-capped as acetate esters (BHE).

Partially deprotonated (10%) triethyl citrate (6.00 g, 0.02 mol) was equipped in a reaction bulb under a nitrogen atmosphere, and 16 g of glycidol (12 equiv) was slowly dropped in the reaction bulb at 75 °C for 4 h. Then, the mixture was stirred at 100 °C for another 4 h to finish the reaction. The product was dissolved in methanol to obtain yellowish viscous BHP. Excess acetic anhydride was mixed with BHP and stirred at 130 °C under a nitrogen atmosphere for 3 h to finish the reaction. The product was dissolved in chloroform. BHE was obtained after washing with sodium hydroxide solution and distilled water, drying with magnesium sulfate and removing water and chloroform with reduced pressure distillation. Figure 1 shows the synthesis of BHE. 

### 2.3. Preparation of PVC Films

PVC paste resin (3.0 g) was mixed with 30 wt %, 40 wt %, 50 wt % or 60 wt % BHE in 60 mL of THF solvent and stirred using a magnetic stirrer for 20 min at 50 °C [17]. The obtained transparent mixtures was poured into glass Petri dishes and dried in a constant-temperature drying box for 48 h to completely remove the residual THF. PVC films were prepared and labeled S1, S2, S3 and S4. Pure PVC and PVC containing 60 phr of DOP were prepared according to the same method and labeled PVC and SD. 

### 2.4. Characterization 

BHP and BHE were characterized with FT-IR spectra and ^1^H and ^13^C nuclear magnetic resonance (^1^H NMR). The FT-IR spectra were investigated using a Nicolet iS10 FTIR (Nicolet Instrument Corp., Madison, WI, USA) Fourier transform infrared spectrophotometer. ^1^H and ^13^C NMR were performed using an AV-300 NMR spectrometer (Bruker Instrument Corp., Karlsruhe, Germany) at a frequency of 400 MHz. CDCl_3_ was used as solvent, and tetrametnylsilane (TMS) was used as an internal standard in the process. The infrared absorption peak of ester groups in plasticized PVC materials was also detected via FT-IR according to the above method.

The thermal stability of PVC materials and plasticizers was investigated using a TG209F1 thermal gravimetric analyzer (TGA) instrument (Netzsch Instrument Corp., Bavaria, Germany) in N_2_ atmosphere (50 mL/min). The heating rate was 10 °C/min.

The plasticizing efficiency of BHE was evaluated by *T_g_*, which was investigated using a dynamic thermomechanical analysis (DMA) Q800 (TA Instruments, New Castle, DE, USA) measurement with a frequency of 1 Hz in a dual cantilever mode under N_2_ atmosphere. The temperature ranged from −20 to 120 °C at a heating rate of 3 °C/min. The plasticizing efficiency of BHE was calculated according to Equation (1) [18,19]:

(1) $$E_{\Delta T_{g}}(\%)=\frac{\Delta T_{g,plasticizer}}{\Delta T_{g,DOP}}\times 100$$

where $E_{\Delta T_{g}}$ = plasticizing efficiency of BHE, and $\Delta T_{g}$ = the reduction in Tg from PVC to plasticized PVC films.

The plasticizing performance of PVC materials was evaluated via tensile strength and elongation at break, which were detected according to GB/T 1040.1-2006 (Beijing, China) and at room temperature using an E43.104 Universal Testing Machine (MTS Instrument Corp., Shenzhen, China) [20]. 

The solvent resistance of PVC materials was investigated according to the American Society for Testing Materials (ASTM D5227) and relevant papers [21,22]. PVC films were weighed and then immersed in distilled water, 10% (v/v) ethanol solution, 30 % (w/v) acetic acid solution and petroleum ether, respectively. The test condition was controlled at 23 ± 2 °C, and the relative humidity was restricted at 50% ± 5%. After 24 h, the solvent-extracted PVC materials were dried and reweighed. The weight loss (WL) was calculated according to Equation (2).

(2) $$WL=\frac{W_{1}-W_{2}}{W_{1}}\times 100$$

where W_1_ is the initial weight of PVC materials, and W_2_ is the final weight of test PVC materials. The extraction loss data were collected using the average value of five test samples. 

The volatility of BHE was evaluated by placing plasticized PVC samples in a convection oven at 70 °C for 24 h and cooling them to room temperature in a desiccator for 2 h. The weight changes were measured before and after the heating.

The acute oral toxicity test was carried out by Ningbo Entry-Exit Inspection and Quarantine Bureau Technical Center according to Chinese National Standards (GB/T 21603-2008) and relevant papers [19,23]. The temperature of the barrier environment was maintained at 22.5–23.7 °C, and the relative humidity was restricted to 45.9–52.7%. The Institute of Cancer Research (ICR) mice were provided by the Zhejiang Experimental Animal Center. The experimental animals were divided into two groups, each of which contained 10 males and 10 females. A dose of 5 g/kg was given to both groups. The two groups were observed 3 h after dosing and once daily thereafter for a total of two weeks. All experimental animals were weighed once a week and sacrificed at the end of the observation period. The cardiac, liver, lung and spleen index of the experimental animals were examined via necropsy.

## 3. Results

**Figure 2:**
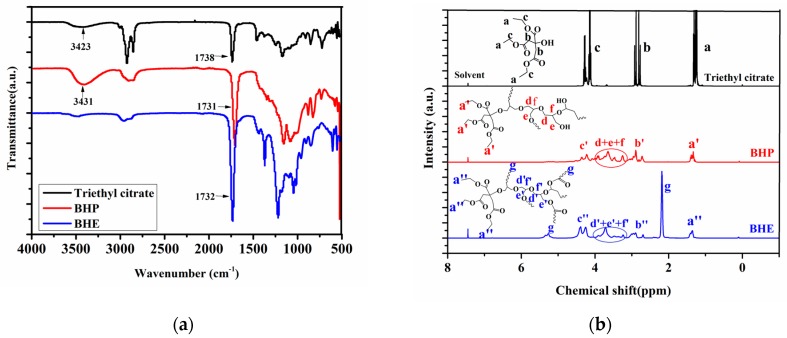

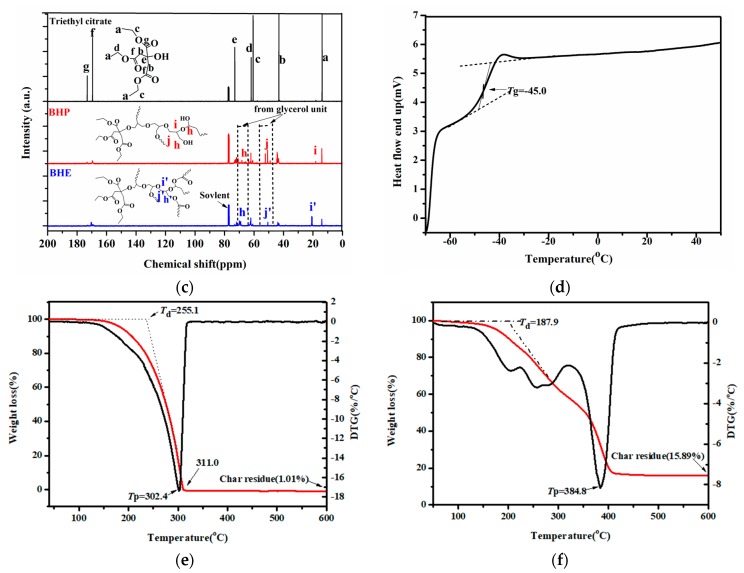
(**a**) FT-IR of triethyl citrate, BHP and BHE; (**b**) 1H NMR of triethyl citrate, BHP and BHE; (**c**) 13C NMR of triethyl citrate, BHP and BHE; (**d**) DSC curves of BHE; (**e**) TGA and DTG curves of DOP; (**f**) TGA and DTG curves of BHE.

BHE was first synthesized through a facile one-pot reaction. Figure 2a presents the FT-IR spectra of BHE, BHP and triethyl citrate. The facile one-pot reaction is divided into two stages. The first stage is to synthesize BHP via ring-opening polymerization of glycidol and triethyl citrate. As seen from the FT-IR of triethyl citrate, the broad –OH group FT-IR band can be observed at 3423 cm^−1^; after the ring-opening polymerization, the broad –OH group FT-IR band appears at 3431 cm^−1^ and is stronger than that of triethyl citrate, indicating that the ring-opening polymerization occurred and that more hydroxyl groups were generated [24]. The second stage is the esterification of BHP and acetic anhydride. As seen from the FT-IR of BHE, after the esterification, the characteristic absorption peak of the –OH group disappears, and the C=O group FT-IR band is stronger than BHP, which illustrates that esterification was complete [12]. In order to further track the facile one-pot reaction, ^1^H NMR and ^13^C NMR of BHP and BHE were investigated; they are shown in Figure 2b,c. In the 1H NMR of triethyl citrate, the strong peak (Figure 2a) appearing at 1.25 ppm is associated with methyl protons, the peak (Figure 2b) appearing at 2.89 ppm is attributed to protons of methylene next to the carbonyl group, and the two strong peaks (Figure 2c) appearing at 4.1–4.4 ppm are assigned to protons of different methylenes next to ether groups. When compared with ^1^H NMR of triethyl citrate, new peaks (Figure 2d−f) appear at 3.2–4.0 ppm and correspond to protons of a different methylene derived from glycerol units in the ^1^H NMR of BHP, illustrating that BHP was obtained [25]. In the ^1^H NMR of BHE, a new strong peak (Figure 2g) at 2.4 ppm is assigned to protons of methyl groups next to a carbonyl group, illustrating that esterification was finished. Figure 2c shows the ^13^C NMR of triethyl citrate, BHP and BHE. As seen from the ^13^C NMR of triethyl citrate, the peak (Figure 2a) at 14.14 ppm corresponds to carbons of methyl groups, and the peak (Figure 2b) appearing at 43.40 ppm is assigned to carbons of methylene groups next to carbonyl groups. The signals (Figure 2c,d) appearing at 60.88 ppm and 62.22 ppm are attributed to carbons of methylene groups connected to methyl groups. The signal (Figure 2e) of carbons connected to hydroxyl groups appears at 73.30 ppm. The signals (Figure 2f,g) at 169.77 and 173.40 ppm correspond to carbons of carbonyl groups [25,26]. Compared with the ^13^C NMR of triethyl citrate, new signals appear in the ^13^C NMR of BHP; the new signals (Figure 2i,j) appearing at 21.50 ppm and 31.10 ppm are assigned to carbons next to ether groups, and the peaks at around 64 ppm and 72 ppm are attributed to carbons connected to hydroxyl groups that are derived from glycidyl units, indicating that the ring-opening polymerization was complete. The ^13^C NMR of BHE shows similar results to that of BHP, except the peak (Figure 2i) at 21.50 ppm appears stronger than for BHP; this peak is assigned to carbons next to ether groups, illustrating that the esterification was finished and BHE was successfully synthesized. 

Figure 2d shows the DSC curves of BHE; the *T_g_* of BHE is −45 °C. The thermal degradation process of DOP and BHE was performed via TGA. The excellent thermal stability of the plasticizers has a positive effect on the heat resistance and stability of plasticized PVC materials. In addition, the high thermal stability of plasticizers causes low volatility, which keeps the properties of PVC materials stable for a long time. Figure 2e,f show the TGA and DTG curves of DOP and BHE. In Figure 2e, only one thermal rapid degradation stage can be found for DOP, and the thermal degradation temperature (T_d_) and the temperature of peak thermal degradation rate (T_p_) are 255.1 °C and 302.4 °C. There are two degradation stages for BHE; T_d_ and T_p_ for BHE are 187.9 °C and 284.8 °C. It is obvious that the thermal stability of DOP is better than that of BHE in the low-temperature range. However, DOP was almost completely degraded at 311 °C and only produced 1.01% char residue at 600 °C, while BHE stopped degrading at 384.8 °C and produced 15.89% char residue at 600 °C. A high char residue yield is a good indicator of the improvement of the thermal stability of BHE [27,28,29].

Low-toxicity or non-toxic plasticizers can be used in a wide range of PVC products, such as food packaging materials, toys and medical equipment, to replace reproductive toxic phthalate plasticizers [30]. Test results of oral toxicity of BHE are shown in Table 1. The acute toxicity of BHE was tested using a predictive model for non-toxicity. A single dose of 5 g/kg was administered by gavage to the experimental animals. The results indicated that no significant treatment-related signs of toxicity or behavioral changes were present in any of the experimental rats during the 14-day observational period after a single dose of BHE. The experimental groups displayed no histopathological changes in the liver, kidney, heart, spleen, ovaries, testes or intestines relative to the control group. Acute oral toxicity results indicated that BHE had toxic doses of 5g/kg, suggesting that BHE is non-toxic.

**Figure 3:**
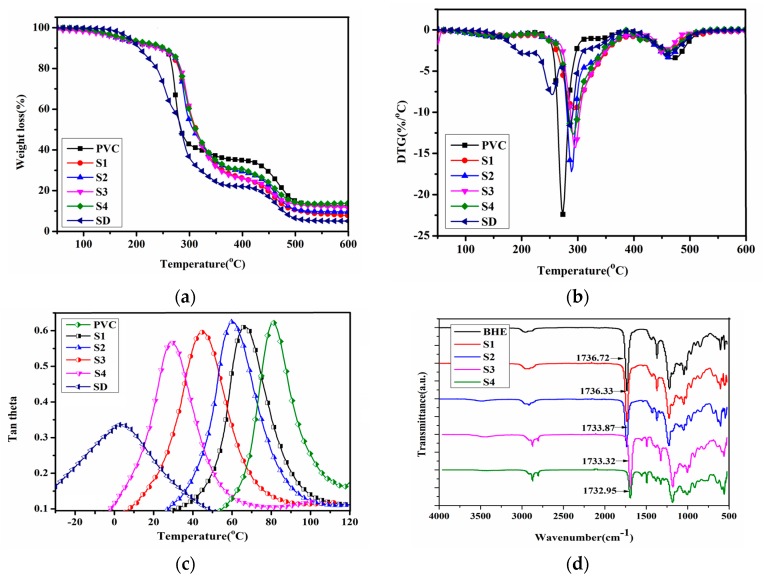
(**a**) TGA curves of PVC materials; (**b**) DTG curves of PVC materials; (**c**) DMA curves of PVC materials; (**d**) FT-IR of PVC materials.

Normally, PVC thermally degrades easily as a result of structural defects such as head-to-head units, tertiary chlorines at branched carbons and chlorine atoms adjacent to internal double bonds [11]. The addition of plasticizer changes the thermal stability of PVC materials. The performance of PVC plasticized with DOP was reported in our previous study, and the results showed that DOP severely decreased the thermal stability of plasticized PVC materials, which limits the application of plasticized PVC materials in products that require a high temperature [2,12]. In order to improve the thermal stability of PVC materials and suppress the dehydrochlorination degradation of PVC, generally, heat stabilizers (including organic heat stabilizers such as epoxidized soybean oil and inorganic heat stabilizers such as zinc stearate and calcium stearate) are blended with PVC materials [31,32]. Figure 3a,b show the TGA and DTG curves of all PVC materials. The thermal degradation data, including thermal degradation temperature (*T_g_*), the temperature of peak heat release rate (T_p_) and char residue, are summarized in Table 2. It can be clearly observed that there are two pyrolysis processes, including dechlorination and cross-linking of C=C bonds to form char residue for all PVC materials [1]. The addition of DOP decreased the thermal stability of PVC materials, which is consistent with our previous study [2,12]. The T_d_ of SD is 217.0 °C lower than that of PVC. Char residue for SD is lower than for PVC, and only 5.07% of its initial mass is left. For PVC plasticized with BHE samples, thermal stability is improved with the further addition of BHE because BHE is thermally stable in the high-temperature range and produces more char residue than DOP; the char residue has the effect of covering and isolating heat sources on PVC materials, which further suppresses thermal degradation. 

The plasticizing efficiency of plasticizers is closely related to their chemical structure and physical properties. Plasticizers with a lower relative molecular mass, less of a ring-like chemical structure, more ether chains and short alkyl chains have higher plasticizing efficiency [33,34]. The addition of plasticizer can decrease the interaction force between polymer molecules and increase the mobility of polymer chains. Plasticizing efficiency is usually evaluated by *T_g_*. The polymer presents flexibility at temperatures above *T_g_* and becomes hard below *T_g_*. The lower *T_g_* of plasticized PVC materials shows excellent plasticizing properties. In our case, the *T_g_* of PVC and plasticized PVC material was detected via DMA. Figure 3c shows tan theta peaks of PVC and PVC materials plasticized with a different plasticizer content. The *T_g_* values are summarized in Table 2. The tan theta peaks correspond to *T_g_*. It can be observed that there is only one tan theta peak for these PVC materials, which indicates that PVC and plasticizers, including DOP and BHE, are completely compatible with PVC, and there is no phase separation between PVC and plasticizers because strong hydrogen bonds between H atoms in PVC and carbonyl group in BHE result in better miscibility and only one *T_g_* [11]. The *T_g_* of PVC is 82.5 °C, and the *T_g_* decreased with the addition of BHE. When the content of BHE was 60 wt % of PVC, the *T_g_* of PVC material reached 28.3 °C, while the *T_g_* of the SD sample reached −1.2 °C, which illustrates that the plasticizing efficiency of BHE was lower than that of DOP. The EΔ*T_g_* value of BHE reached 64.8%. 

**Figure 4:**
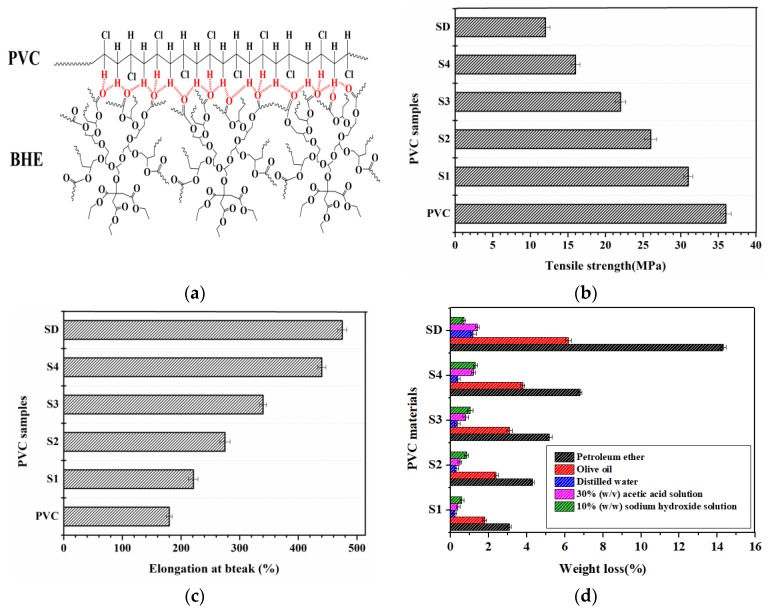
(**a**) Formation of hydrogen bonds; (**b**) tensile strength of PVC materials; (**c**) elongation at breaks of PVC materials; (**d**) solvent extraction resistance of plasticizers.

In order to further investigate the plasticizing mechanism and the relationship between miscibility and hydrogen bonds in a PVC/plasticizer system, the FT-IR of PVC plasticized with different content of BHE was detected. Figure 3d shows the FT-IR spectra, and Figure 4(1) shows the hydrogen bonding formation between PVC and BHE molecules. Polar Cl atoms and H atoms on the same C atoms as Cl can readily form hydrogen bonds with carbonyl oxygen on BHE molecules [11,35]. As seen from Figure 3d, the infrared absorption peak of the carbonyl group in BHE shifts from 1736.72 cm^−1^ to 1732.95 cm^−1^ when the content of BHE reaches 60 wt % of PVC, which demonstrates that the hydrogen bond was formed, as seen from Figure 4a. The formation of hydrogen bonds results in the expected miscibility of PVC and BHE, which further causes an excellent plasticizing effect. 

The mechanical properties of plasticized PVC materials were also investigated because the excellent compatibility of PVC and plasticizer causes a high elongation at break and low tensile strength. Figure 4b,c show the tensile strength and elongation at break of all PVC materials. The results show that the addition of BHE led to a significant decrease in tensile strength and an increase in elongation at break. The elongation at break increased from 180% (PVC) to 440% (S4), and tensile strength decreased from 36 MPa (PVC) to 17 MPa (S4), which suggests that BHE has an excellent plasticizing effect on PVC. However, tensile strength and elongation at break for SD are 12 MPa and 475%, indicating that the plasticizing effect of DOP is better than that of BHE. 

**Figure 5:**
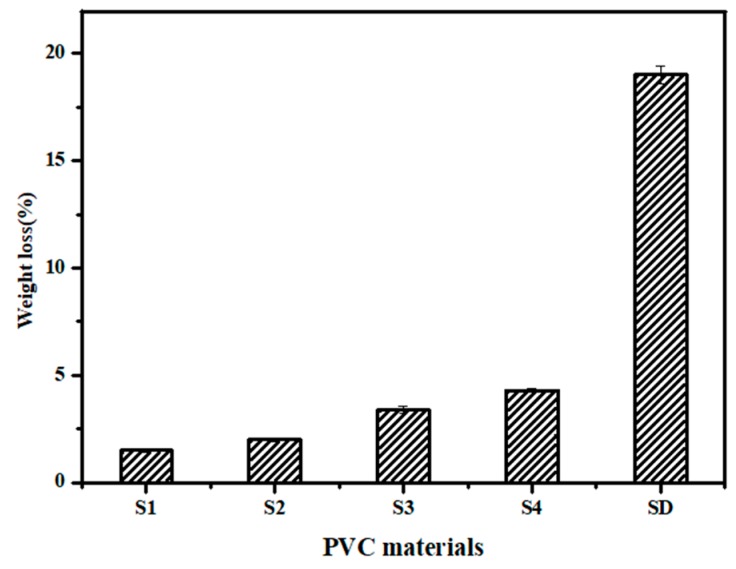
Volatilization resistance of plasticizers.

Plasticizers with a high molecular weight and a high branching degree usually have better solvent extraction resistance, which has a positive effect on keeping the properties of PVC materials long-term stability and decreases the risk of plasticizer toxicity in humans. In our case, solvent extraction resistance of BHE in different solvents and volatilization resistance were detected and compared with those of DOP. The results are shown in Figure 5. It is clearly shown that PVC materials lost more weight with the addition of BHE. SD samples lost more weight than S4 samples, which illustrates that the solvent extraction and volatilization resistance of BHE are better than those of DOP because BHE has a higher molecular weight, a higher branching degree and more polar groups than DOP and has a strong interaction with PVC. The results show that BHE can be used as a non-toxic plasticizer with excellent migration resistance to replace DOP.

## 4. Conclusions

The synthesis of biomass-based hyperbranched poly(ether)s end-capped as acetate esters (BHE) was accomplished in a facile one-pot reaction of triethyl citrate, glycidol and acetic anhydride. BHE was completely miscible with PVC, though it presented a lower plasticizing effect than DOP (EΔ*Tg* value of BHE reached 64.8%). BHE showed more solvents and volatilization resistance than DOP. Furthermore, PVC-plasticized BHE showed better thermal stability than PVC and PVC-plasticized DOP materials. The cardiac, liver, lung and spleen indices of experimental animals did not show abnormal changes in the acute oral toxicity test. The acute oral toxicity test indicated that BHE had toxic doses of 5 g/kg, suggesting that BHE is non-toxic. This excellent performance makes BHE extremely attractive as bio-based, non-toxic and feasible plasticizers for flexible PVC materials with various applications in different fields.

## Figures and Tables

**Table 1:** Results of acute oral toxicity test.

Gender	Numbers	Weight ( $\bar{X}$ ± Std (g)			Number of Deaths	Mortality (%)
		0 day	7 days	14 days		
Male	10	19.2 ± 0.68	24.1 ± 0.56	28.41 ± 1.40	0	0
Female	10	19.5 ± 1.01	23.12 ± 1.43	26.23 ± 1.62	0	0

**Table 2:** TGA and DSC data for PVC materials.

Samples	*T*_d_ (°C)	*T*_p_ (°C)	Char Residue (%)	*Tg* (°C)
PVC	263.9	272.9	9.59	82.5
S1	272.4	289.9	9.58	69.1
S2	276.8	292.4	11.74	58.4
S3	278.2	293.7	12.10	44.6
S4	280.6	295.0	13.80	28.3
SD	217.0	286.6	5.07	−1.2
